# Biofilm Formation on Dental Implant Surface Treated by Implantoplasty: An In Situ Study

**DOI:** 10.3390/dj8020040

**Published:** 2020-05-06

**Authors:** Francesco Azzola, Andrei Cristian Ionescu, Marco Ottobelli, Nicolò Cavalli, Eugenio Brambilla, Stefano Corbella, Luca Francetti

**Affiliations:** 1 Department of Biomedical, Surgical and Dental Sciences, IRCCS Galeazzi Orthopedic Institute, University of Milan, 20161 Milan, Italy; cavalli.nicolo@gmail.com (N.C.); Stefano.Corbella@unimi.it (S.C.); luca.francetti@unimi.it (L.F.); 2 Oral Microbiology Laboratory, Department of Biomedical, Surgical and Dental Sciences, University of Milan, 20161 Milan, Italy; andreiionescu_40@hotmail.com (A.C.I.); marco.ottobelli@unimi.it (M.O.); eugenio.brambilla@unimi.it (E.B.); 3 Institue of Dentistry, Department of Oral Surgery, I.M. Sechenov First Moscow state Medical University, 119146 Moscow, Russia

**Keywords:** peri-implantitis, implantoplasty, biofilm, scanning electron microscopy, confocal laser scanning microscopy

## Abstract

Peri-implantitis is a biofilm-related disease whose characteristics are peri-implant tissues inflammation and bone resorption. Some clinical trials report beneficial effects after implantoplasty, namely the surgical smoothening of the implant surface, but there is a lack of data about the development of the bacterial biofilm on those smoothened surfaces. The aim of this study is to evaluate how implantoplasty influences biofilm formation. Three implants with moderately rough surfaces (control) and three implants treated with implantoplasty (test) were set on a tray reproducing the supra- and sub-gingival environment. One volunteer wore this tray for five days. Every 24 h, plaque coverage was measured and, at the end of the period of observartion, the implant surfaces were analyzed using scanning electron microscopy and confocal laser scanning microscopy. The proportion of implant surface covered with plaque was 65% (SD = 7.07) of the control implants and 16% (SD = 0) of the test implants. Untreated surfaces showed mature, complex biofilm structures with wide morphological diversity, and treated surfaces did not show the formation of mature biofilm structures. This study supports the efficacy of implantoplasty in reducing plaque adhesion and influencing biofilm formation. These results can be considered a preliminary proof of concept, but they may encourage further studies about the effects of implantoplasty on biofilm formation.

## 1. Introduction

Oral implant rehabilitations can be affected by a biological complication called peri-implantitis that causes the loss of support bone. The term peri-implantitis is introduced by Levignac [1] to describe the infectious disease that creates peri-implant osteolysis. Then, Mombelli^2^ et al. [2], in a study on the bacterial colonization affecting implants with and without bone resorption, concludes that peri-implantitis is a “specific infection site that has much in common with periodontitis”. In 1993, the European Workshop of Periodontology [3] distinguished peri-implant mucositis from peri-implantitis: the first one is defined as “an inflammatory reaction reversible soft tissue surrounding an implant subjected to functional load” and the second one as “inflammation of the tissues with peri-implant bone loss of support”. Consequently, the term peri-implantitis, as confirmed in the consensus report of workgroup 4 of the 2017 World Workshop on the Classification of Periodontal and Peri-Implant Diseases and Conditions [4], should be used specifically for the inflammatory processes mediated by bacteria that lead to the formation of peri-implant pockets and the resorption of bone tissue around the implant. This specification also implies that bone remodeling following the insertion of an implant must be distinguished from bone resorption due to the action of microorganisms.

Several authors [5,6,7] recognize that the steps of biofilm development on implant and natural teeth are similar. Regarding the composition of the biofilm, no significant differences between natural roots and titanium implants could be highlighted [2,3,4,5,6,7,8]. 

Similar to periodontitis, different therapeutic approaches are indeed proposed for peri-implantitis [9]: non-surgical therapy, access flap, resective surgery, and regenerative surgery; these treatments are associated with various decontamination methods of the implant surface, such as air flow, saline solution, laser, curettes, ultrasonic cleaning. However, despite the variety of approaches and decontamination methods, no generally accepted treatment protocols curing peri-implantitis are available [9,10]. A beneficial effect is nevertheless reported in some clinical trials [11,12] as a result of a modification of the implant surface, commonly called implantoplasty, in association with a resective surgical approach or with a combined resective and regenerative surgical approach [13,14,15,16]. Implantoplasty [17] consists in the removal of implant threads and achieving a smooth implant surface by using rotary instruments. Its purpose is firstly to remove the most external layer of titanium, alleged to be contaminated with bacteria, creating an uncontaminated surface, and secondly to reduce the implant roughness in order to make its surface less retentive for plaque. However, there is a lack of studies investigating the actual effect of this procedure on the plaque development.

The first aim of our study is therefore to verify if an implantoplasty procedure is able to reduce biofilm formation on implant surfaces in situ, namely the human mouth, through a macroscopic quantitative measurement. An additional aim is to observe if implantoplasty could influence biofilm morphology; this qualitative analysis of the biofilm morphology is made using scanning electron microscopy (SEM, 1 specimen/surface type) and confocal laser scanning microscopy (CLSM, 1 specimen/surface type)

## 2. Materials and Methods

### 2.1. Specimens Preparation

The samples consisted of six implants (BTI Biotechnology Institute 2011. San Antonio, 15, 5° 01005 Vitoria-Gasteiz; Álava): four implants were 11.5 long and 3.8 mm in diameter, the remaining two were 8 mm long and 3.8 mm in diameter, all implants were covered with a moderately rough acid-etched surface. Half of the samples (two implants measuring 11.5 mm and one measuring 8 mm) were treated with implantoplasty, as it was described by Romeo et al. [11]; an experienced operator, wearing a 4× magnification loupes, planished the threads and smoothed the implant surface through a sequence of burs working at 15,000 rpm under constant irrigation. The bur sequence included a diamond, 30-µm particle size, egg-shaped bur followed by a diamond, 15-µm particle size, egg-shaped bur (Komet, Gerb. Brasseler GmbH, Lemgo, Germany), Arkansas burs and, finally, silicon polishers (Shofu Inc., Kyoto, Japan). Specimens were then carefully rinsed with distilled water to remove debris from the surfaces. 

### 2.2. Volunteer Selection and Tray Preparation

A volunteer (23 years old woman) was selected according to the following inclusion criteria: absence of gingivitis or periodontal disease, non-smoker and non-user of mouthwashes in the previous 6 months, absence of any prosthetic or implant rehabilitation. We obtained full informed consent (signed form) by the subject.

**Figure 1:**
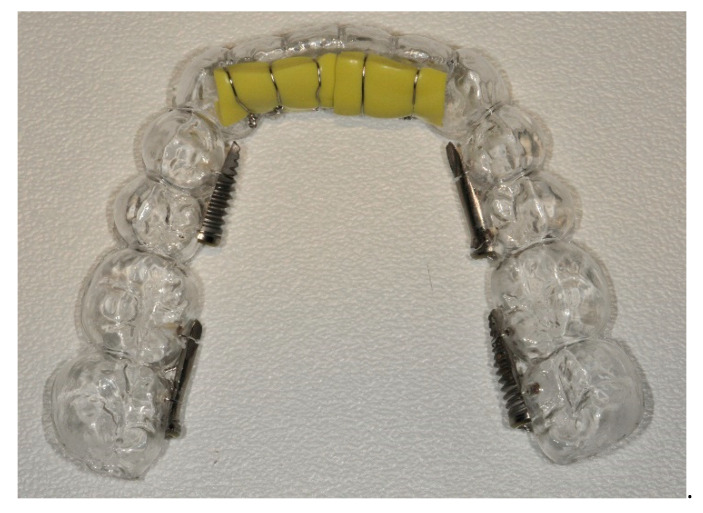
Customized thermoplastic mouth tray.

A customized thermoplastic mouth tray was obtained on the inferior arch of the volunteer using a plaster model. All six implants were separated vertically along the major axis using diamond cutting discs (Kerr Dental, Orange, CA, USA). The implants were then fixed on the lingual surfaces of the tray by means of orthodontic ligatures (0.1 mm stainless steel wire). The two 8-mm implants were placed in correspondence of the incisors, and the four 11.5-mm implants were positioned in correspondence of molars and premolars, alternating the position of the implants treated with implantoplasty (right side: mesial treated, distal untreated; left side: mesial untreated, distal treated). A layer of vinylpolysiloxane (VPS) impression material (Imprint™ II Garant™ Regular Body, 3M ESPE, Seefeld, Germany) was cast over the two implants in the incisal position to reproduce the subgingival microaerophilic environment. The material was allowed to set and was then detached, repositioned and fixed into position using orthodontic ligatures in order to cover the implants without sealing (Figure 1).

### 2.3. In Situ Procedures

Seven days before the beginning of the experiment, the volunteer received professional oral hygiene, and since that moment until the end of the observation she was instructed to only use toothpaste without fluoride or antimicrobial agents. The tray was consecutively worn by the volunteer during the observation period (5 days) and it was removed three times a day, after meals, for regular at-home oral care. The tray was not cleaned in any way and the volunteer was instructed not to touch implant surfaces while removing or repositioning the tray. 

### 2.4. Macroscopic Evaluation

During the observation period, every 24 h, the four exposed implants were photographed using a standardized orthogonal frame and a digital reflex camera (D300S. NIKON CORPORATION, Tokyo, Japan) equipped with zoom lens (AF-S 105 mm f/2.8 G ED VR Micro.NIKON CORPORATION, Tokyo, Japan) and ring flash (Mecablitz 15 MS-1 Digital Flash METZ CORPORATION, Zirndorf, Germany) always using the same magnification ratio. A virtual grid of 150 squares was then applied on the obtained images by image processing. The quantification of implant surface covered by plaque was obtained by counting the number of squares containing plaque and the percentage of the surface covered with plaque was thus obtained. 

Means and standard deviations for surface plaque coverage were calculated; despite the extremely low number of specimens, statistical analysis was performed by using Student’s t post-hoc test [18] to assess significant differences, setting *p* < 0.05. The methodology was reviewed by an independent statistician.

### 2.5. Microscopic Evaluation

At the end of the observation period, the tray was removed and immediately placed into a Petri dish containing sterile PBS at 37 °C. The ligatures were cut and all implants were removed using sterile tweezers without touching the surface. Specimens were then subjected to microscopic observation.

Specimens were placed in a 2% glutaraldehyde-cacodylate buffered fixative solution for 1 week. The specimens were then transferred to 35%, 50%, 70%, 80%, 85%, 90%, and 95% (*v*/*v*) ethanol solutions, and finally two times in 100% ethanol solutions for 10 min each. Immediately after that, the specimens were critical point dried (Critical-point Dryer, EMS 850, Hatfield, PA, USA), mounted on stubs with conductive glue, sputter coated (JEOL FFC-1100, Tokyo, Japan), and observed using a scanning electron microscope (JEOL FFC-1100, Tokyo, Japan) at magnifications ranging from 10× to 40,000×.

Specimens subjected to CLSM observation were stained using the FilmTracer Live/Dead Biofilm Viability Kit for microscopy (Invitrogen Ltd., Paisley, UK). The fluorescence from stained cells adherent to the samples was observed using a CLSM (Leica TCS SP2, Leica Microsystems, Wetzlar, Germany). Four randomly selected image stack sections were recorded for each biofilm specimen. Confocal images were obtained using a dry 20× objective with a numerical aperture (NA) of 0.7 and digitalized using the Leica Application Suite Advanced Fluorescence Software (LAS AF, Leica Microsystems, Wetzlar, Germany) at a resolution of 2048 × 2048 pixels, with a zoom factor of 1.0. For each image stack section, an average intensity projection (AIP) and three-dimensional (3D) reconstructions were obtained using specific softwares (ImageJ. National Institutes of Health, Bethesda, MD, USA).

## 3. Results

### 3.1. Macroscopic Evaluation

Macroscopic analysis is shown in Table 1. No significant differences were found on both test and control surfaces regarding plaque accumulation comparing plaque accumulation on Day 1 with the beginning of the experiment. Considering surfaces treated with implantoplasty, plaque accumulation was significantly higher on Day 5 compared to Day 1 (*p* = 0.0435) or to baseline (*p* = 0.0153). On control surfaces, a highly significant increase in plaque accumulation was observed from Day 1 to Day 2 (*p* = 0.0009), then a constant increase occurred each day until Day 5. This increase was not significant on Day 3 (compared to Day 2, *p* = 0.3649). Starting from Day 2, a highly significant difference was always found between treated and untreated surfaces. After five days, 65% (SD = 7.07) of control implants’ and 16% (SD = 0) of test implants’ surfaces were covered with plaque.

Plaque Table 1 legend: formation on test (implantoplasty) and control implants, as assessed photographically as surface plaque coverage.

### 3.2. Microscopic Evaluation

**Figure 2:**
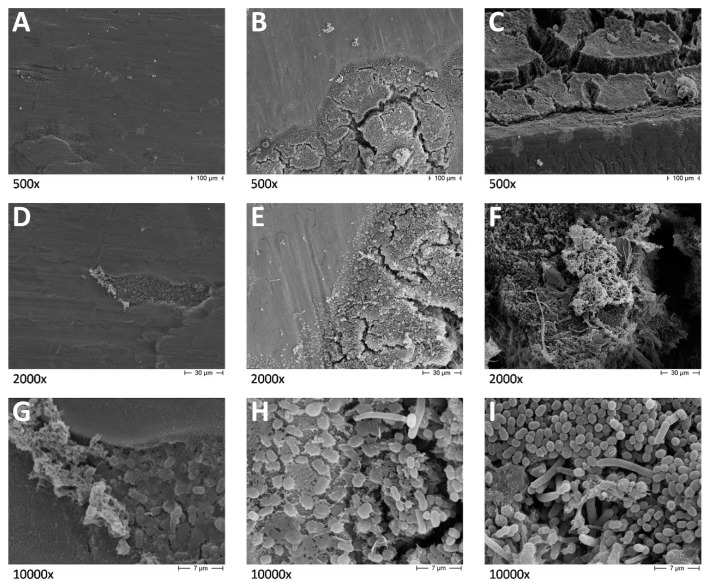
Scanning electron microscopy (SEM) micrographs of test. (**A**) test implant at 500× magnifications; (**B**) bottom part of the test implant at 500× magnifications; (**C**) control implant at 500× magnifications; (**D**) test implant at 2000× magnifications; (**E**) bottom part of the test implant at 2000× magnifications; (**F**) control implant at 2000× magnifications; (**G**) test implant at 10,000× magnifications; (**H**) bottom part of the test implant at 10,000× magnifications; (**I**) control implant at 10,000× magnifications.

**Figure 3:**
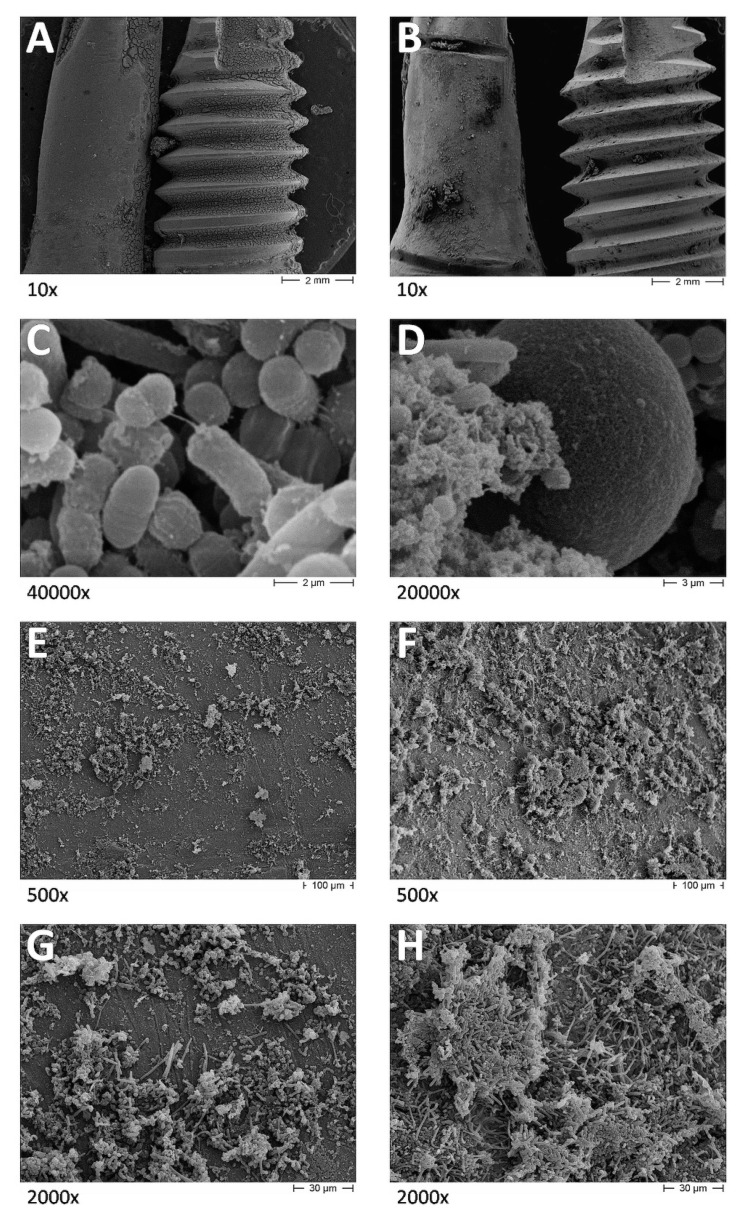
SEM micrographs of control specimens. (**A**) test and control specimens exposed to oral environment at 10× magnification; (**B**) test and control specimens covered by VPS layer at 10× magnification; (**C**) control specimen exposed to the oral environment at 40,000× magnification; (**D**) control specimen covered by VPS layer at 40,000× magnification; (**E**) Test specimen covered by VPS layer at 500× magnification; (**F**) control specimen covered by VPS at 500× magnification; (**G**) test specimen covered by VPS layer at 2000× magnification; (**H**) control specimen covered by VPS at 2000× magnification.

**Figure 4:**
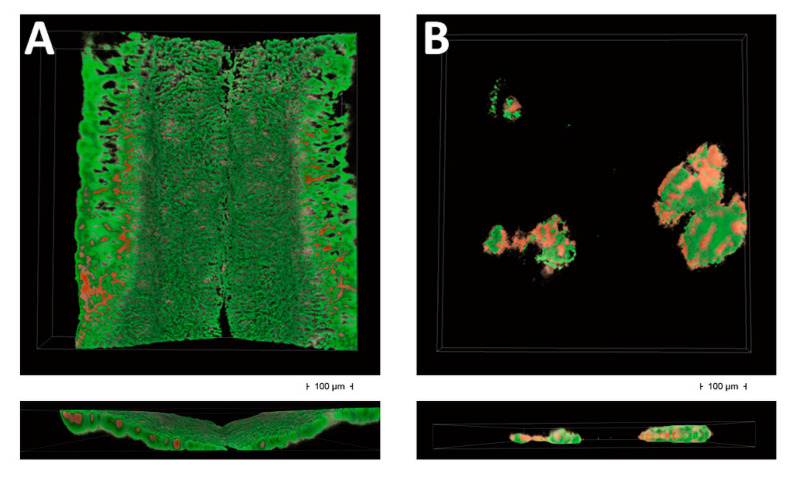
CLSM analysis. Three-dimensional reconstructions of 750 × 750 μm fields from the specimens exposed to the oral environment. Live/Dead stain colors viable microorganisms in green, while the nonviable ones are shown in red. (**A**) space between two threads of a control implant; (**B**) test implant.

SEM (Figure 2 and Figure 3) and CLSM (Figure 4) showed very different plaque structures depending on the surface treatment. Untreated surfaces of uncovered implants showed mature and complex biofilm structures with wide morphological diversity expressing active replication and inter-species communication through pili. On the surfaces treated with implantoplasty, less mature biofilm structures were observed, showing a prevalence of cocci embedded in the extra-cellular matrix. A similar difference was observed between the covered implant surfaces, even if the examined biofilms had a smaller diversity, being mostly composed by bacilli and filamentous microorganisms, possibly as a consequence of the sub-gingival-like conditions created by the silicon envelope.

Figure 2A,D,G shows implant surfaces subjected to implantoplasty at different magnifications. It is evident that the treatment provided a very smooth surface; however, small surface irregularities are still present. The irregularities are used by pioneer bacteria, almost exclusively cocci, as adhesion and colonization sites. A significant amount of extracellular matrix (ECM) production can be better seen in G, protruding from the colonization site towards the upper left corner of the picture and out from the niche, favoring additional bacteria adherence and further biofilm development. Figure 2B,E,H shows implant surfaces subjected to implantoplasty at the bottom part of the specimen. Here, reduced clearance and shear forces favor biofilm development. Biofilm development and maturation can be seen as bacteria progressively colonize the smooth surfaces starting from the bottom part of the specimen. A first layer of pioneer cocci adheres to the surfaces, then immediately start ECM production (E). After that, biofilm develops as a complex multilayered structure in which mainly bacilli and some hyphae can be observed, while the surface disappears under the mature biofilm structures. Figure 2C,F,I show control implant surfaces showing huge biofilm formation, especially in the spaces between threads. Mature biofilm structures having more than 100 μm of thickness in some points can be observed. They are composed of mixed bacterial species, cocci, bacilli and hyphae, embedded in large amounts of ECM. 

These observations indicate that implantoplasty procedures do not completely prevent bacterial adherence and biofilm formation, but biofilm structures are much more easily removed by shear forces and mechanical action of oral structures than conventional implant surfaces.

Figure 3A shows an overview of test and control specimens exposed to oral environment. Biofilm formation is prevalent on surfaces less subjected to shear forces, such as the bottom of the specimen subjected to implantoplasty (that is on the right side of the picture) and the spaces between threads on the conventional implant. Figure 3B shows an overview of test and control specimens covered by the VPS layer. An even biofilm formation can be seen on both surfaces, with the accumulation of debris at some points. Figure 3C,D shows very high magnification micrographs showing biofilm on conventional implant surfaces, exposed to the oral environment or covered by the VPS, respectively. In Figure 3C, a bacterial pilus is connecting a replicating coccus and a bacillus, showing the very complex interactions existing inside oral biofilms. Figure 3D shows cocci and bacilli embedded into huge amounts of ECM growing over the surface of a blastospore. Figure 3E,G shows implant surfaces subjected to implantoplasty and covered by the VPS at different magnifications. A relatively low and even surface colonization can be seen by biofilm structures, which appear less dense and compact than those exposed to the oral environment. Figure 3F,H shows control implant surfaces covered by the VPS at different magnifications. Higher surface colonization by more compact, multilayered structures can be seen in all fields compared to the implantoplasty surfaces. This difference cannot be explained by shear forces or clearance, since both surfaces were protected under the same layer of VPS material.

Figure 4A shows the conventional implant surface, and the space between two threads is shown having a vertical direction. Mature biofilm structures can be observed composed mostly by viable organisms. The reconstruction shows a higher ratio of dead to live microorganisms in the upper parts of the field, which are the layers more exposed to the environment. Figure 4B shows the implantoplasty surface showing isolated microcolonies having a ratio of dead to live microorganisms of about 0.5. Dead cells are mostly fount on the outer layers of the microcolonies. Very initial evidence of confluent growth is shown, even after 5 days, indicating that implantoplasty treatment is able to prevent both microbial adherence and the maturation of biofilm structures.

## 4. Discussion

The aim of this study was to investigate the effect of implantoplasty on biofilm development. Despite its wide use and documented efficacy, alone or in a combined resective/regenerative therapy, in the treatment of peri-implantitis, it has not been adequately investigated from a microbiological point of view.

At first, in vivo studies [19] showed a correlation between plaque adhesion and surface roughness on natural teeth surfaces. Afterwards, it was observed [20] that the same correlation was maintained even if the roughness was created on acetate strips, and, when a similar experiment [21] was conducted on titanium, rough abutments harbored 25 times more bacteria than standard abutments in accordance with the results of the present study. Similar results in terms of plaque accumulation were obtained by Zitzmann et al. [22] and in 2014 by Schwarz et al. [23]. In a review of the literature by Renvert et al. [24], the author concluded that “based on the limited data available, there is no evidence that implant surface characteristics can have a significant effect on the initiation of peri-implantitis”. This statement seems to be confirmed by an in vivo study by Albouy et al. [25], in which, however, the progression of the peri-implant lesion seems to be influenced by the roughness of the surface possibly predisposed by a different biofilm quantity and composition.

The results described in the present paper, indeed, show significant differences in terms of plaque accumulation and the biofilm morphology of both supra-gingival and sub-gingival biofilm models. In fact, the differences between biofilm formation on a conventional implant rough surface and a smoothened one are not only in the amount of bacteria. In the first case, it was observed that a biofilm forms mature structures over time, while over the smooth surface it does not. The biofilm was seen developing on treated surfaces starting from the hard-to-clean peripheral areas. This observation could suggest that the possibility of cleaning surfaces treated with implantoplasty plays a major role in preventing plaque accumulation and biofilm formation.

Moreover, SEM observations of the untreated implants reveal that the most colonized surfaces are those situated in the deepest depressions between threads. Since implantoplasty eliminates both the surface roughness and threads it is not clear how much each of them may contribute to biofilm development.

John and colleagues [26] evaluated the plaque removing effectiveness of a rotating titanium brush in comparison with the cleaning procedures with steel curettes. The study was conducted on titanium discs expressly fabricated with a sandblasted and acid etched surface, and the discs were splinted to trays worn inside the mouth similarly to our study but on a different location (palatally, at a 1-mm distance from the mucosa). That study, in spite of having a different aim, confirms that the use of splinted specimens in volunteers’ mouths could be an effective in situ method to investigate the correlation between surface characteristics and biofilm formation. 

However, the present investigation has to be considered a preliminary study because of the sample size restricted to only one volunteer. Furthermore, the efficacy of implantoplasty treatment was compared to only one rough implant surface type (surface-etched, coated with nano-sized calcium-phosphate particles). A comparison with other types of implant surfaces is mandatory in future studies. Another possible limit is represented by the ideal conditions in which implantoplasty was performed in this study, not taking into account the difficulties that normally characterize the surgical access to an osseointegrated implant. In order to corroborate implantoplasty for the treatment of peri-implantitis, potentially adverse consequences, such as the dispersion of titanium filing, the over-heating of the peri-implant tissues, and implant structural weakening, should also be evaluated.

The results observed in this experiment should encourage new studies to investigate the efficacy of implantplasty in reducing plaque accumulation and influencing plaque structure in both aerobical and microaerophilic environments.

This study, through the replication of supra- and sub-gingival conditions, supports the efficacy of implantoplasty in influencing biofilm maturation and in reducing plaque accumulation. These results can be considered a preliminary proof of concept, but they may encourage further studies about the effects of implantoplasty on peri-implantitis therapy.

## Figures and Tables

**Table 1:** Plaque formation.

	Control Right	Control Left	Test Right	Test Left	Control	Test
	Plaque Presence/150 Squares, (%)				Mean % (±SD)	
Day 1	6 (4.00%)	4 (2.67%)	5 (3.33%)	0 (0.00%)	3.35 (0.92)	1.65 (2.33)
Day 2	37 (24.67%)	47 (31.33%)	9 (6.00%)	12 (8.00%)	27.95 (4.74)	7.00 (1.41)
Day 3	50 (33.33%)	50 (33.33%)	15 (10.00%)	15 (10.00%)	33.30 (0.00)	10.00 (0.00)
Day 4	93 (62.00%)	56 (37.33%)	18 (12.00%)	18 (12.00%)	49.65 (17.47)	12.00 (0.00)
Day 5	105(70.00%)	90 (60.00%)	24 (16.00%)	24 (16.00%)	65.00 (7.07)	16.00 (0.00)
